# Mice with cleavage-resistant N-cadherin exhibit synapse anomaly in the hippocampus and outperformance in spatial learning tasks

**DOI:** 10.1186/s13041-021-00738-1

**Published:** 2021-01-25

**Authors:** M. Asada-Utsugi, K. Uemura, M. Kubota, Y. Noda, Y. Tashiro, T. M. Uemura, H. Yamakado, M. Urushitani, R. Takahashi, S. Hattori, T. Miyakawa, N. Ageta-Ishihara, K. Kobayashi, M. Kinoshita, A. Kinoshita

**Affiliations:** 1 School of Health Sciences, Graduate School of Medicine, Kyoto University, Kyoto, Japan; 2 Department of Neurology, Kyoto University Graduate School of Medicine, Kyoto, Japan; 3 Department of Neurology, Shiga University of Medical Science, Seta-Tsukinowa-Cho Otsu, Shiga, 520-2192 Japan; 4 Division of Systems Medical Science, Institute for Comprehensive Medical Science, Fujita Health University, Toyoake, 470-1192 Japan; 5 Division of Biological Sciences, Department of Molecular Biology, Nagoya University Graduate School of Science, Nagoya, 464-8602 Japan; 6 Department of Pharmacology, Graduate School of Medicine, Nippon Medical School, Tokyo, 113-8602 Japan

**Keywords:** N-cadherin, ADAM10, Synapse, Hippocampus, Working memory

## Abstract

N-cadherin is a homophilic cell adhesion molecule that stabilizes excitatory synapses, by connecting pre- and post-synaptic termini. Upon NMDA receptor (NMDAR) activation by glutamate, membrane-proximal domains of N-cadherin are cleaved serially by a-disintegrin-and-metalloprotease 10 (ADAM10) and then presenilin 1(PS1, catalytic subunit of the γ-secretase complex). To assess the physiological significance of the initial N-cadherin cleavage, we engineer the mouse genome to create a knock-in allele with tandem missense mutations in the mouse N-cadherin/Cadherin-2 gene (*Cdh2* ^R714G, I715D^, or GD) that confers resistance on proteolysis by ADAM10 (GD mice). GD mice showed a better performance in the radial maze test, with significantly less revisiting errors after intervals of 30 and 300 s than WT, and a tendency for enhanced freezing in fear conditioning. Interestingly, GD mice reveal higher complexity in the tufts of thorny excrescence in the CA3 region of the hippocampus. Fine morphometry with serial section transmission electron microscopy (ssTEM) and three-dimensional (3D) reconstruction reveals significantly higher synaptic density, significantly smaller PSD area, and normal dendritic spine volume in GD mice. This knock-in mouse has provided in vivo evidence that ADAM10-mediated cleavage is a critical step in N-cadherin shedding and degradation and involved in the structure and function of glutamatergic synapses, which affect the memory function.

## Introduction

N-cadherin is a key cell adhesion molecule required not only for dendrite arborization and axon guidance during development, but also for post-developmental generation, maintenance, and remodeling of synapses [1–6]. N-cadherin abounds in active zones of immature synapses and at the periphery of mature synapses [7], indicating its propensity to change its location as well as functional role between synaptogenesis and mature synapses. Trans-synaptic contact mediated by the N-cadherin/catenin complex is a determinant of neuro-transmission and spine morphology, and reciprocally, synaptic activity modulates the expression, conformation, targeting, degradation and proteolytic cleavage of N-cadherin [8–13].

As with other synaptic adhesion molecules, activity-dependent and -independent proteolytic cleavage of surface-presented N-cadherin attenuates a trans-synaptic adhesive force, facilitating synaptic remodeling [14–16]. A representative case is that of ADAM10-meditated cleavage at post-developmental excitatory synapses, followed by PS1/gamma-secretase-mediated (epsilon) cleavage, which releases a nuclear-targeting fragment CTF2 from N-cadherin [17–19].

Inhibition of the ADAM10-meditated initial cleavage increases the steady-state level of the full-length N-cadherin. Notably, stabilization of N-cadherin by inhibition of ADAM10 activity increases the spine head volume and the recruitment of GluR1into the postsynaptic compartment, thereby regulating the function of AMPA receptors at the hippocampal excitatory synapses [30]. However, most of these findings are based on pharmacological experiments with cultured neurons or brain slices, and in vivo relevance has never been tested. Thus, we set up to test the impact of ADAM10-mediated N-cadherin cleavage on animal behavior. To analyze this, on the basis of our previous studies [20], we created a line of knock-in mice that express N-cadherin with a mutation resistant to ADAM10-mediated cleavage (GD mice).

Here, we demonstrate that the GD mice show higher fidelity in a working memory-dependent task than WT mice in the radial maze test. In the fear-conditioning test, GD mice show a tendency to enhanced freezing. This behavioral change is accompanied by higher complexity in the tufts of thorny excrescence and significantly higher synaptic density, significantly smaller PSD area, and normal dendritic spine volume in the CA3 region of the hippocampus. Our mouse model helps address the open questions as to the physiological and pathological impacts of the cleavage-defective N-cadherin on synapse morphology and transmission, learning and memory, and other brain functions.

## Results

### Generation of a knock-in allele encoding the ADAM10-resistant mutant of N-cadherin

**Fig. 1:**
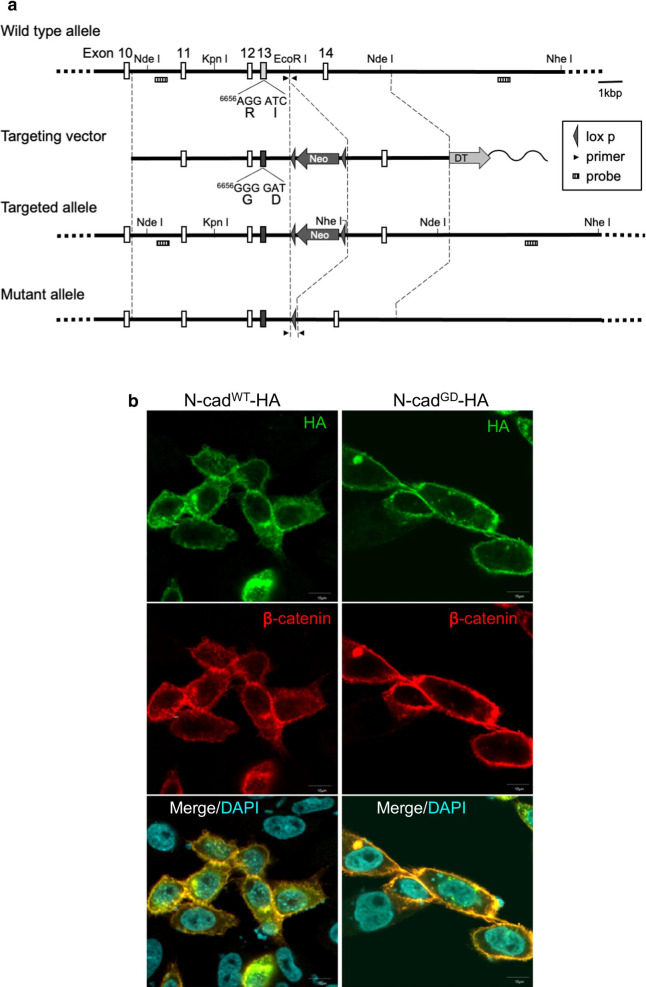
Introducing ADAM10-uncleavable GD mutations into *N-cadherin*/*Cdh2* in the mouse genome, and testing the mutant distribution in cultured cells. **a** Genomic DNA map of the mouse *Cdh2* gene locus, mutant design, and targeting strategy. The gene targeting vector was designed to introduce tandem missense mutations (^6656^AGG ATC for ^714^RI → GGG GAT for GD) in the exon 13 of the mouse *Cdh2* gene. The targeting vector was introduced into C57BL/6-derived ES cells by electroporation. Neomycin-resistant clones were screened for homologous recombination with genomic Southern blotting using two probes, and one such clone was injected into ICR blastocysts. The resulting chimera mice were intercrossed with C57BL/6 N mice. The floxed neomycin-resistant cassette was removed from the allele by intercrossing the F1 mice with a line of CAG-Cre driver mice of C57BL/6 strain. The mutant and wild type alleles were discriminated routinely by PCR genotyping, using a pair of primers (arrowheads). See “Methods” for details. **b** Representative immunofluorescence images of FLAG-tagged, WT and GD mutant N-cadherin expressed in CHO cells. Their distribution patterns on ER, Golgi apparatus, and plasma membrane (green), as well as colocalization with endogenous β-catenin (red), were comparable. Nuclear DNA is stained with DAPI (cyan)

Our previous in vitro analysis showed that a tandem substitution mutation near the transmembrane domain of mouse N-cadherin (R714G/I715D, hereafter GD; Fig. 1a) confers resistance on ADAM10 [20]. To assess the physiological significance of ADAM10-mediated cleavage of N-cadherin in the brain, we designed a targeting vector to replace exon 13 of the mouse *Cdh2* gene with the GD mutant exon, by homologous recombination (Fig. 1a). After gene targeting of an ES cell line (Bruce4) derived from C57BL/6 J strain, we screened Neomycin-resistant clones for a GD allele (*Cdh2*^R714G, I715D^) by southern blotting, PCR, and sequencing of the genomic DNA (data not shown). Chimeric mice generated through blastocyst injection of one of Cdh2R714G, I 715D/ + ES cell clones into C57BL/6 J embryos were bred with WT mice to yield heterozygous (Cdh2R714G, I715D/ +) mice with the genetic background of C57BL/6 J. The floxed Neomycin-resistant gene cassette was removed by breeding with CAG-Cre driver C57BL/6 J mice. After confirming mendelian inheritance of the GD allele, we consistently compared homozygous GD mutant (*Cdh2*^R714G, I715D/R714G, I715D^) and WT (*Cdh2*^+/+^) male littermates born from heterozygous parents.

### A mutant N-cadherin that is uncleavable by ADAM10 is relatively stable on the cell surface

**Fig. 2:**
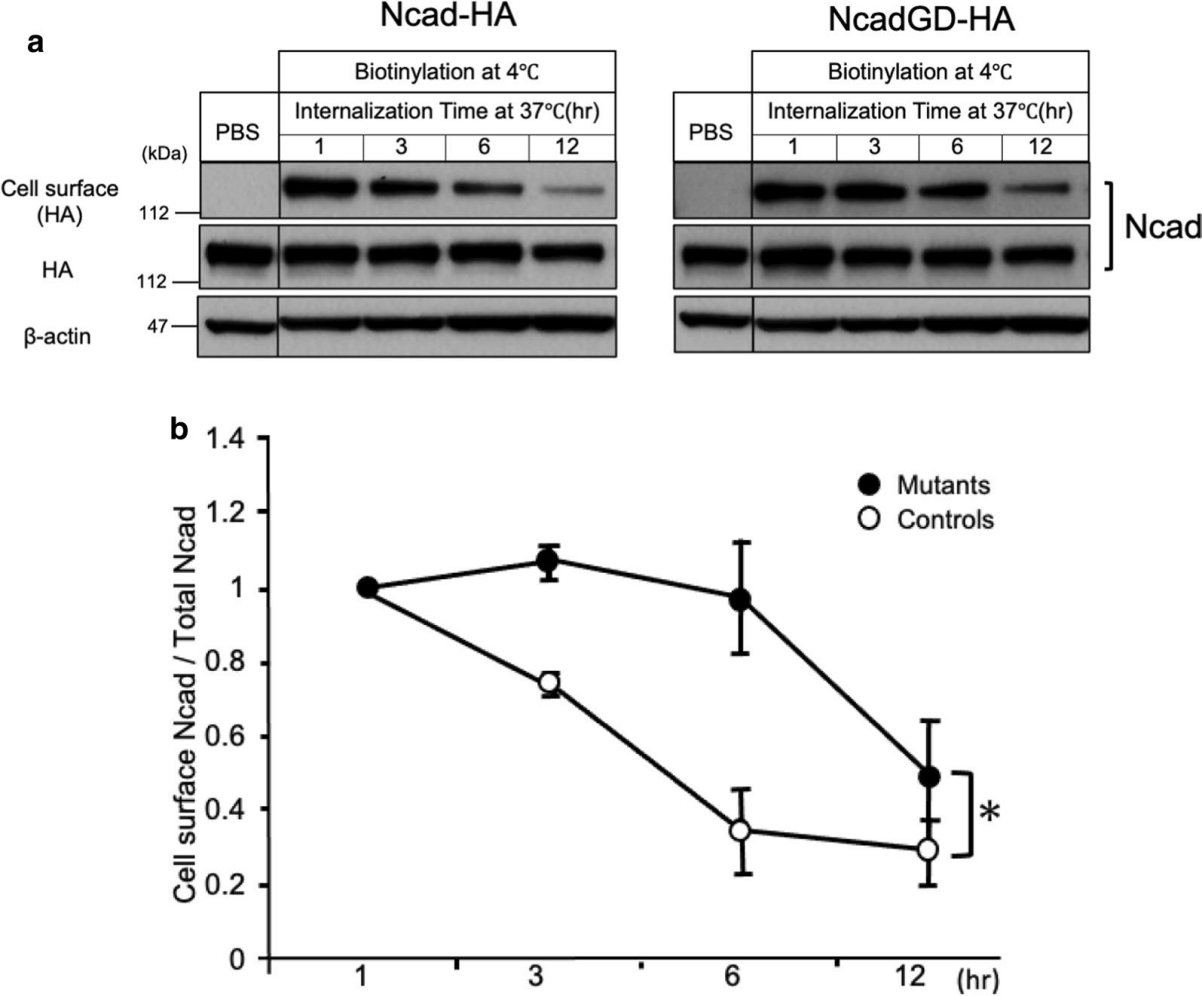
GD mutant N-cadherin on the plasma membrane is relatively stable. **a** Representative immunoblot data from cell surface protein bio-tinylation analysis for HA-tagged, WT (left) or GD mutant (right) N-cadherin expressed in CHO cells. Surface bio-tinylation pulse-labeling followed by serial immunoblot assay for the HA epitope shows that cell surface fraction of GD and WT N-cadherins decay within hours, while the levels of total N-cadherins remain unchanged. It is to be noted that the cell surface fraction of GD N-cadherin was significantly more stable than that of WT. (B) (Top) Densitometric quantification of surface biotinylation assay demonstrated that GD mutant (●) on the plasma membrane decayed significantly more slowly than WT (○). (n = 3, 3, p = 0.0241, ANOVA)

First, to assess the resistance of GD-mutant cadherin to ADAM10-mediated cleavage, we expressed wild type (WT) or GD mutant N-cadherins, each tagged with HA epitope, in CHO cells. Their distribution patterns to the endoplasmic reticulum (ER), Golgi apparatus, and plasma membrane were largely comparable (Fig. 1b). On the other hand, surface biotinylation pulse-labeling followed by serial immunoblot assay for the HA epitope revealed that the cell surface fraction of GD N-cadherin was significantly higher than that of WT, indicative of a longer half-life (~ 12 h vs. ~ 4 h; Fig. 2a, b). These in vitro data indicate that GD mutation prolongs the retention of the full-length N-cadherin on the plasma membrane, demonstrating that ADAM-10 mediated cleavage of N-cadherin affects the level of cell-surface N-cadherin.

### GD mutation does not show obvious difference in the content of major synapse proteins in synaptosome

Previous reports demonstrated that systemic and brain-selective null mutant mice of N-cadherin, respectively, exhibit severe systemic and neural anomalies [2, 4, 22], indicating a requirement of N-cadherin for development. In contrast, GD mice grow and breed normally, suggesting that the perturbation of the N-cadherin cleavage at the site does not cause obvious defects in embryonic and post-natal development.

**Fig. 3:**
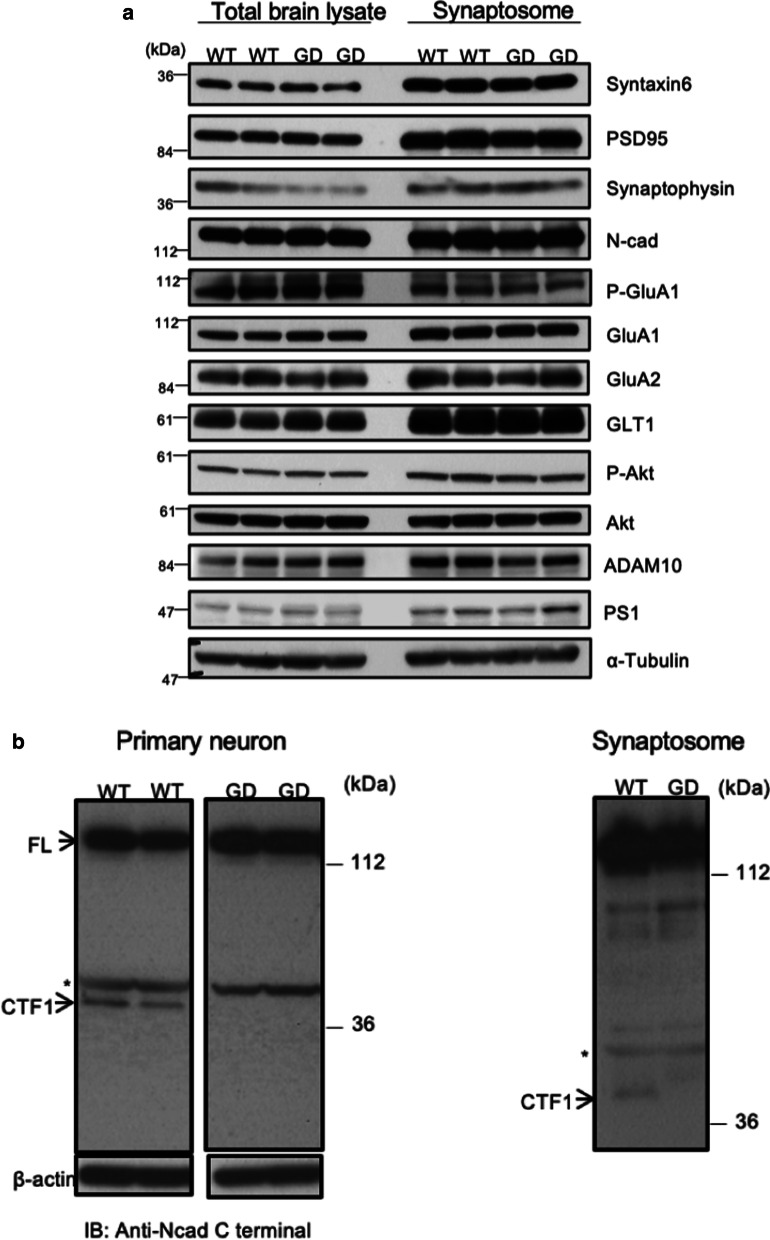
Protein profiles of GD mouse brain is normal but for the absence of CTF1. **a** Representative immunoblot of endogenous proteins related to glutamatergic synapse and/or N-cadherin in the total brain lysate and a synaptosomal fraction from GD and WT mice. No recognizable difference was found in syntaxin 6, PSD95, synaptophysin, full-length N-cadherin, phospho-GluA1, GluA1, GluA2, GLT-1, phospho-AKT, AKT, α-tubulin). **b** Immunoblot detection of endogenous N-cadherin with an antibody against a C-terminal region in the total lysates of primary cultured cerebrocortical neurons from WT and GD mice. While the full-length form (FL, 130 kDa) was detected both in WT and GD samples, the cytoplasmic fragment CTF1 (45 kDa) was detected only in those from WT, but not in those from GD mice (left). Similar results were obtained from the synaptosomal fraction (right). Non-specific bands appeared around 50 kDa (*) and above

Since cleaved ectodomain fragments of N-cadherin were barely detectable from the brain tissue samples, we examined the cytoplasmic fragment CTF1 in primary cultured cerebrocortical neurons and synaptosomal fractions from WT and GD mice. An antibody against the carboxyl terminus of N-cadherin detected native full-length N-cadherin and CTF1 in WT neurons, while CTF1 was not detected in GD neurons (Fig. 3b). CTF2, a g-secretase-mediated cleavage product of CTF1, was barely visible in this setting due to proteosomal degradation [20]. These data indicate the ADAM10-mediated cleavage-resistance of N-cadherin^R714G, I715D^*in vivo*, in line with the enhanced stability of exogenously expressed N-cadherin^R714G, I715D^ in the surface biotinylation assay (Fig. 2a, b) and previous in vitro studies [20, 21].

Next, we investigated whether the content of excitatory synapse-related proteins is altered in the total brain lysate and the synaptosomal fraction of the GD mice. Immunoblot analysis showed no obvious difference between steady-state levels of full length N-cadherins (GD and WT), and most of major post/pre/peri-synaptic proteins and activity-regulated proteins in those mice; *i.e.*, ADAM-10, PS1, GluA1, GluA2, AKT, phospho-AKT, syntaxin 6, synaptophysin, and GLT-1 in synaptosome. (Fig. 3a).

### Behavioral profile of GD mice is largely normal, except for correct responses in a spatial working memory task

Next, we examined whether the insertion of GD mutation alters mouse behavior. As a means of unbiased functional screening for possible alterations in the GD mouse brain, we systematically conducted a learning memory test at 4 months of age. Male GD and WT littermates (n = 12, 9) were comparable in most of the physical and behavioral indices measured, but significantly differed in hippocampus-dependent tests and body weight, as detailed below (Additional file 1: Fig. S1–S4).

**Fig. 4:**
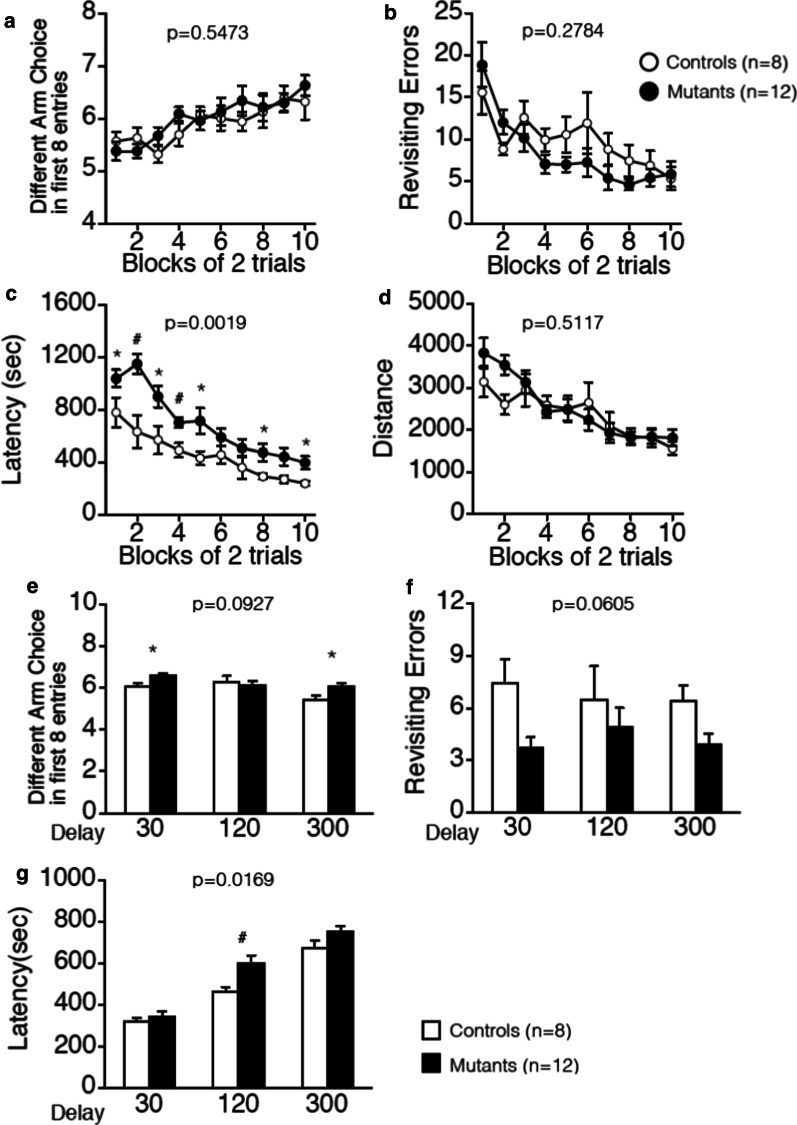
Improvement of working memory performance of GD mice in the eight-arm radial maze test. Spatial learning was tested in the eight-arm radial maze test on 4 months WT (□; n = 9) and GD (■; n = 12) mice. **a**The number of different arm choices in the first eight entries, **b** the number of revisiting errors, **c** the latency to acquire eight pellets and **d** and the distance traveled, are presented. **e**–**g** Results of the eight-arm radial maze test with delays of 3, 120, 300 s. The number of different arm choices in the first eight entries (**e**), the number of revisiting errors (**f**) and latency (**g**) were presented. One WT mouse died after trials. The p-values indicate genotype effect in two-way repeated measures ANOVA (**a**–**g**) or one-way ANOVA (**e**–**g**, for each delay time). Values are means ± SEM

GD mice exhibited an unexpected phenotype in a spatial working memory task using 8-arm radial maze (Fig. 4a–g). The time spent for the acquisition of the reward from the opening of the gates was significantly longer in GD mice (Fig. 4c, g). Intriguingly, however, the revisiting errors after intervals of 30 and 300 secs were significantly less in GD mice than in WT (Fig. 4e, f), indicating that GD mice execute a working memory-dependent task with higher fidelity than WT mice. A similar trend, albeit statistically insignificant, was observed consistently in the probe test of the Barnes maze test, where GD mice tended to reduce the latency to find the escape hole and the number of errors made (Additional file 1: Fig. S3).

**Fig. 5:**
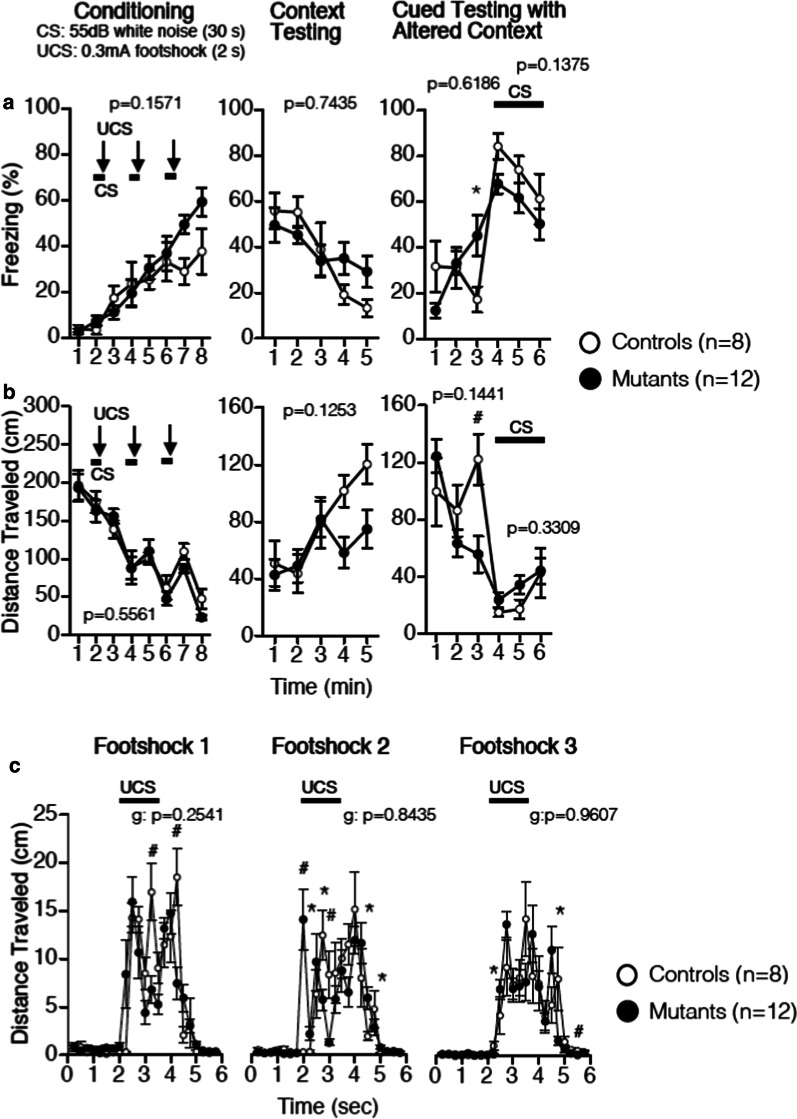
Fear conditioning test in GD mice. **a** Freezing (%) in the conditioning (left panel), context test (middle panel), and cued test (right panel). **b** Distance traveled (cm) in the conditioning (left panel), context test (middle panel), and cued test (right panel). **c** Distance traveled immediately after foot-shocks in the conditioning phase. The p-values indicate genotype effect in two-way repeated measures ANOVA (**a**–**c**). *p < 0.05, **p < 0.01 (one-way ANOVA for each time point). Values are means ± SEM. WT □; n = 8, GD ■; n = 12

In the fear conditioning test, GD mice showed a tendency for enhanced freezing (an index of associative memory) in the late phases of fear memory acquisition on Day 1 (Fig. 5a, b, left) and of re-exposure to the shock box (without tone cue or foot shock) on Day 2 (Fig. 5a, b, middle). Interestingly, in the cued testing in the altered context on Day 2, mutants showed enhanced freezing even before CS (Fig. 5a, b, right). This aberrant behavior could be interpreted as ‘generalization’. Both wild-type and mutant mice reacted to unconditioned stimuli, although the time course of reaction was slightly different (Fig. 5c). Results of behavioral tests are summarized in Additional file 1: Table S1.

### Hippocampal CA3 pyramidal neurons in GD mice develop anomalous synapses

**Fig. 6:**
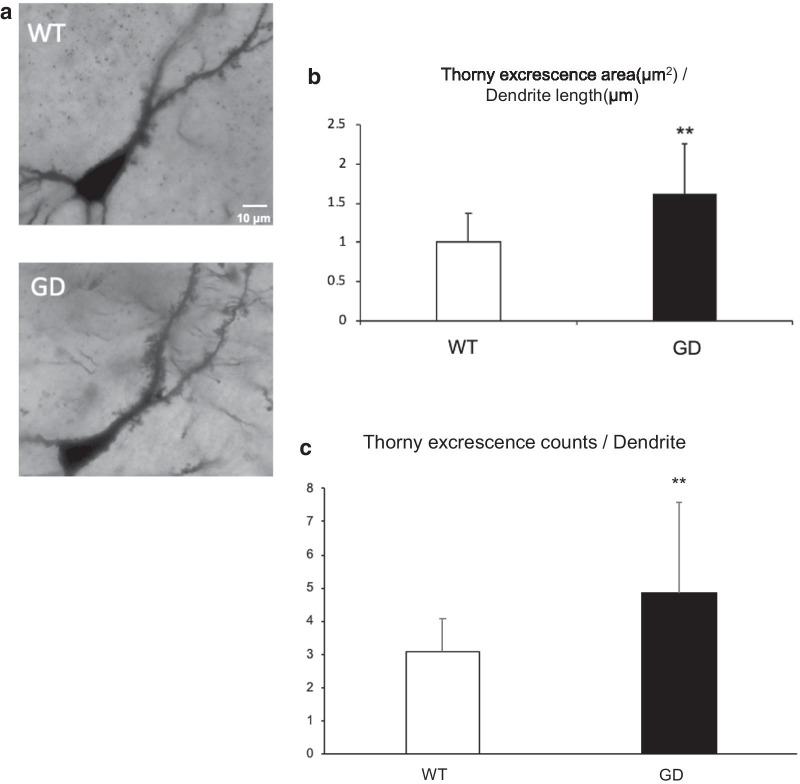
GD mice exhibit synapse anomalies in CA3. **a** Bright field micrographs of Golgi stained brain slices from the stratum lucidum of the CA3 region of the hippocampus. Note the complexity in the tufts of thorny excrescence. **b** The graph shows that the area of thorny excrescence per unit length of dendrite is significantly higher in GD mice. WT; n = 12, GD; n = 15, ** p = 0.0063, t-test. **c** Thorny excrescence counts per dendrite. ** p = 0.0019, t-test. Data presented as mean + SD

To explore the histological anomalies responsible for the behavioral alterations in GD mice, we conducted morphological analysis. The total weight and macroscopic architecture of the brain are normal in homozygous GD mice (Additional file 1: Fig. S4). Given the abundance of *Cdh2* mRNA in the hippocampus, the highest in the CA3 pyramidal neurons, followed by the dentate gyrus granule cells (http://mouse.brain-map.org/experiment/show/79632275), and CA3 being deeply involved in a number of memory and hippocampal learning processes,we first examined the large specialized synapses between them. Golgi stain of the stratum lucidum of the CA3 region revealed significantly higher complexity in the tufts of thorny excrescence (Fig. 6a), which receive mossy fiber inputs from the granule cells. Statistical analysis revealed that the thorny excrescence area (Fig. 6b) and tuft counts (Fig. 6c) were significantly increased in dendrites of GD mice. For comparison, we also examined the synapse density by Golgi staining of the neurons in CA1 region, but found less evident increase in this area (Additional file 1: Fig. S4c).

**Fig. 7:**
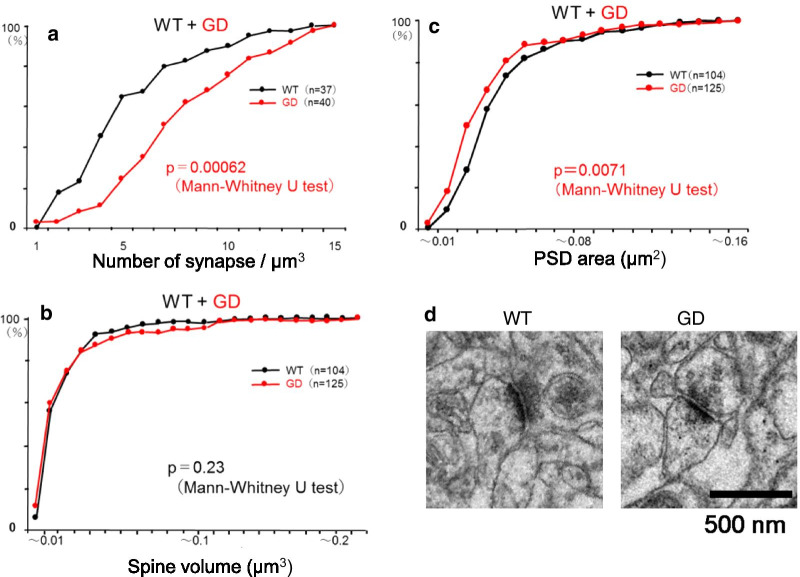
GD mice exhibit synapse anomalies in CA3. **a** Cumulative histogram of asymmetrical synapse density in the stratum radiatum of the CA3 region of the hippocampus. Synapse density measured by the dissector method was significantly higher in GD mice (median value, 7 vs. 4, n: number of images examined. n = 37, 40, p = 0.00062, Mann–Whitney U-test). **b** Cumulative histogram of dendritic spine volume of asymmetrical synapses in the same region. Dendritic spine volume measured with ssTEM/3D reconstruction method was comparable between GD and WT mice (median value, 0.015 vs. 0.015, n: number of spines examined. n = 104, 125, p = 0.23, Mann–Whitney U-test). **c** Cumulative histogram of PSD area of asymmetrical synapses in the same region. PSD area measured with ssTEM/3D reconstruction method was significantly smaller in GD mice (median value, 0.030 vs. 0.035, n: number of spines examined. n = 104, 125, p = 0.0071, Mann–Whitney U-test). **d** Representative images of asymmetrical synapses at CA3 region observed by electron microscopy

Thereafter, we focused on the synapses formed between associational/commissural fibers and pyramidal neurons in the stratum radiatum of the CA3 region, to conduct serial section transmission electron microscopy (ssTEM) for 3D morphometry (Fig. 7). The density of asymmetrical (mostly glutamatergic) synapses measured using the dissector method was significantly higher in GD mice than WT mice (Fig. 7a: median value, 7 vs. 4, p = 0.00062, Mann–Whitney U test). The dendritic spine volume was comparable (Fig. 7b: median value, 0.015 vs. 0.015, p = 0.23, Mann–Whitney U test), whereas PSD area was significantly smaller in GD mice (Fig. 7c, d: median value, 0.030 vs. 0.035, p = 0.0071, Mann–Whitney U test). These findings, along with the Golgi stain results, indicate anomalies in synaptogenesis and/or remodeling of at least two types of synapses into CA3 pyramidal neurons of GD mice.

### The associational/commissural fiber-pyramidal neuron synapses in CA3 exhibit marginal transmission anomalies

**Fig. 8:**
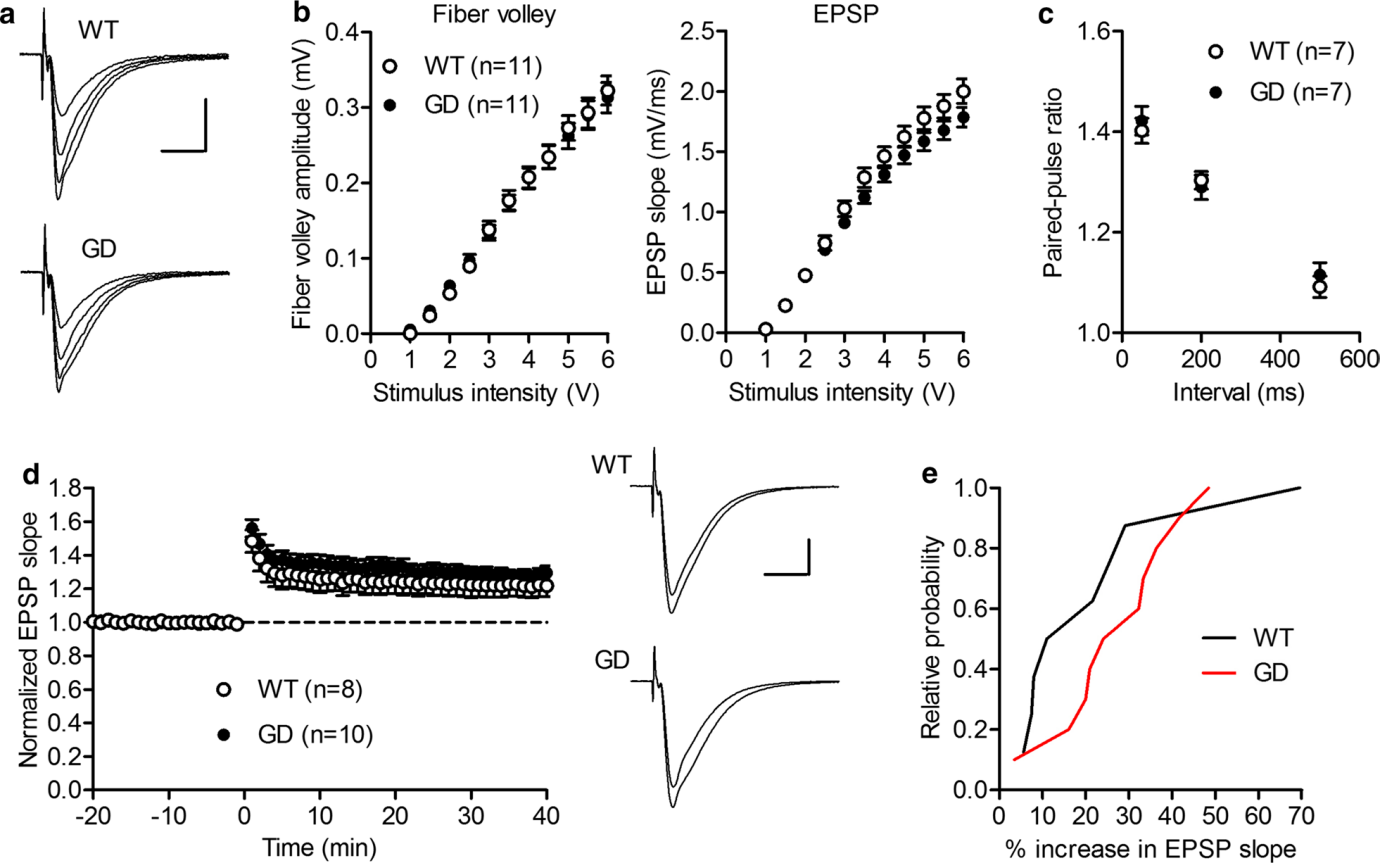
Marginal anomalies of A/C fiber-pyramidal cell synaptic transmission in CA3. **a** A/C fiber-pyramidal cell synaptic potentials evoked at the stimulus intensities of 2, 3, 4 and 5 V in WT and GD mice. Scale bar: 10 ms, 1 mV. **b** Dependence of fiber volley amplitude (left) and EPSP slope (right) on stimulus intensity. Statistically significant interaction between genotype and stimulus intensity was observed for EPSP slopes (two-way repeated measure ANOVA: genotype, F_1,20_ = 2.02, p = 0.1711; stimulus intensity, F_10,200_ = 767.49, p < 0.0001; genotype × stimulus intensity; F_10,200_ = 3.2, p = 0.0008). **c** Normal paired-pulse facilitation of EPSP slopes. **d** Mild augmentation of LTP in the mutant mice. High-frequency stimulation (HFS) was delivered at time 0. Sample traces are averages of 30 consecutive EPSPs during baseline and 30 to 40 min after HFS. **e** Cumulative relative probability distributions of the magnitude of LTP measured at 30 to 40 min after HFS. The number (n) of data represents the number of slices in this figure

To explore possible functional alterations in the synapses formed between associational/commissural (A/C) fibers and pyramidal neurons in the stratum radiatum of the CA3 region, we conducted an electrophysiological recording of field excitatory postsynaptic potentials (EPSPs), using acute hippocampal slices from GD and WT mice. Indices of basal synaptic transmission (*i.e.*, fiber volley amplitude and field EPSP slope) were largely comparable between GD and WT slices (Fig. 8a, b). However, the dependence of the synaptic response on the stimulus intensity was altered, with a trend of reduced EPSP slopes at higher stimulus intensities in GD mice (Fig. 8b). Paired pulse-ratio was not significantly different between GD and WT slices (Fig. 8c). While high-frequency stimulation induced stable long-term potentiation (LTP) in both genotypes, the magnitude of LTP tended to be larger in GD slices (Fig. 8d, e). We also examined the basal synaptic transmission in the CA1 region and found no significant difference between the genotypes (Additional file 1: Fig. S6).

## Discussion

In this study, we generated the first knock-in mouse model in which N-cadherin is systemically replaced into an ADAM10-uncleavable form. In contrast to the previous reports of N-cadherin knock-out mice, showing severe systemic and neural anomalies [2, 4, 22], GD mutant mice in our study grow and breed normally, but exhibit a set of unique phenotypes including (1) enhanced fidelity (or reduced error rate) in a spatial working memory task, and (2) synapse anomaly (more distribution density, less PSD area) in the CA3 regions.

N-cadherin-mediated cell/synapse adhesion is augmented by the cytoplasmic anchorage to the actin cytoskeleton via p120/β/α-catenins and terminated by the proteolytic cleavages, which release extracellular and cytoplasmic fragments [23, 24]. The relative stability of GD mutant N-cadherin on the cell surface (Fig. 2a, b) and the absence of the cytoplasmic fragment CTF1 (Fig. 3b) indicate that the turnover of N-cadherin might be compromised. A previous report by another group demonstrated that the expression of a stable β-catenin mutant augments N-cadherin-mediated synaptic adhesion in vivo, without significantly affecting synaptic transmission and LTP [25, 26]. Interestingly, in the mouse model, N-cadherin stabilization slows synapse remodeling and causes behavioral inflexibility in reversal learning and memory extinction, and drug addiction [25, 26]. The phenotype of GD mice is concordant as to the normal synaptic transmission and LTP. Interestingly, behavioral change similar to ‘inflexibility’in associative learning and memory was observed in GDmice, i.e., excessive freezing in the late phases of fear memory acquisition and of re-exposure to the context (Fig. 5a, b). Interestingly, in the cued testing in the altered context on Day 2, mutants showed enhanced freezing even in the pre-CS period (Fig. 5a), indicating the occurrence of ‘fear generalization’, which has been attributed to hippocampal CA3 and dentate gyrus [27, 28]. Thus, the behavioral change observed in GD mice, i.e. better working memory and fear generalization, might be closely related to synapse anomaly in their CA3 region, observed in the present study. In the current study, 3 learning tests were chosen for unbiased screening for possible functional alteration of GD mice brain. However, to investigate “inflexibility”, a more rigorous examination with additional behavioral tests, such as reversal learning or extinction learning, is necessary. These are the limitations of the present study and issues related to future challenges.

Conditional knockout of ADAM10 in post-natal mouse brain perturbs N-cadherin cleavage, LTP, and dendritic spine morphology (i.e., reduced density, stubby shape), and causes seizure [29]. Pharmacological inhibition of ADAM10 results in spine enlargement in vitro and in vivo [30]. These cases contrast sharply with the quasi-normal phenotype of GD mice with normal-sized dendritic spines (Fig. 6a). Given that ADAM10 cleaves not only N-cadherin, but also many other synaptic substrates that modulate spine shape and adhesion to the presynaptic counterparts [31], the phenotypic difference is attributed to the pleiotropy of ADAM10. On the other hand, spine enlargement caused by GD mutant expression (in addition to the endogenous N-cadherin) [30] may be interpreted as a phenotype caused by simple N-cadherin excess [13], because spine volume is demonstrated to be normal in GD mice, which express normal level of full-length N-cadherin (Fig. 3a). We performed a cell-based experiment to demonstrate whether the levels of N-cadherin GD trans-dimer are higher than those of wild-type or not. The result was not significant (Additional file 1: Fig. S5).

Dendritic spine volume and PSD area, two principal morphometric indices that reflect the excitatory synapse activity, are in close correlation [32–34]. Intriguingly, however, CA3 regions of GD mice contain densely disproportionate synapses with small PSD area on normal-sized spines (Fig. 7b–d). The mechanism underlying this anomalyis unclear, but there are a few possibilities. (1) Circuit immaturity: Stabilized N-cadherin may confer resistance on activity-dependent competitive elimination and pruning [35, 36]. (2) Adaptive compensation (homeostatic scaling): Too many excitatory inputs into a given neuron may suppress maturation of individual synapses [37]. (3) Active zone confinement: stabilized N-cadherin may expand “non-active” synaptic contact around the active zone [38]. These mechanisms are not mutually exclusive. For example, stabilized cadherin might cause higher synapse density (disturbance in activity-dependent competitive elimination), leading to excessive excitatory inputs into a neuron and the compensatory mechanism inhibiting maturation of synapses and finally limiting PSD area. Moreover, the previous report demonstrated that mature neuron can upregulate spine and synapse number to compensate the lost input [39], indicating that reduced PSD size could lead to increased spine number. Given that activity-dependent N-cadherin upregulation contributes to post-epileptic rewiring in the CA3 region [40], similar disproportion and/or high-density synapse anomalies may constitute the pathology.

## Conclusions

Overall, this study has provided in vivo evidence that ADAM10-mediated cleavage is a critical step in N-cadherin shedding and degradation at glutamatergic synapses, which alters hippocampal synapse morphology and animal behaviors related to spatial working memory and contextual fear memory. The unique mouse model established in this study will serve as a tool to explore the roles of N-cadherin processing in physiological synapse remodeling, and the pathological dysregulations which accompany epilepsy, Alzheimer’s disease, anxiety disorders and other neurological disorders.

## Methods

### Animal experiments

Animal experiments were approved by institutional review committees and conducted in accordance with the regulations for the care and use of animals at Kyoto University and Nippon Medical School. We compared male littermates raised in the same cages, unless otherwise noted.

### Generation and establishment of a line of *Cdh2*^R714G, I715D^ knock-in mice

We designed a targeting vector that spans a 9.6 kb region of the mouse the *Cdh2* gene to replace six bases in exon 13 from ^6656^AGGATC to GGGGAT. After electroporation of the linearized vector into C57BL/6 mouse-derived Bruce 4 embryonic stem cells and selection of neomycin-resistant clones, we verified homologous recombination by Southern blotting and PCR (5′ side probes; Fw-GATGCTGCTAACAGATGACTACAGA, Rv-AAAGGTACTGACAATAGGGCTCATA and 3′ side probes; Fw-TCTCAAAGACTCCTATTGCTGTTCT, Rv-GTGTCTATAAGCTCCCATCAATGTC) of the genomic DNA. After blastocyst injection of the recombinant clones, we obtained chimera mice, which were crossbred with transgenic mice that ubiquitously express Cre-recombinase (*CAG-Cre*, C57BL/6, RBRC01828). Through backcrossing with C57BL/6 J mice for more than two generations, we verified the removal of the neomycin-resistant gene cassette and the *CAG-Cre* allele by Southern blotting and PCR, and Mendelian transmission of the *Cdh2*^R714G, I715D^ allele. We bred heterozyzous *Cdh2*^R714G, I715D/+^ mice to generate homozygous (*Cdh2*^R714G, I715D/R714G, I715D^; GD) and control (*Cdh2*^+/+^; WT) littermates for experiments. The line has been deposited with the Center for Animal Resources and Development, Kumamoto University (ID 2027).

### Genotyping

DNA from tail snips was purified using the automatic DNA isolation system PI-50(KURABO, Japan). Mouse *Cdh2* gene were amplified using the following primers: Forward = CCA CTT CTA AGC ATG CAG GT; Reverse = AAT GAC TCC TAT TTG AGC ACA.

### Behavioral analyses

We conducted behavioral tests with male littermates during 3–5 months of age, using an established protocol [41]. The behavioral tests were conducted in the following order: general health and neurological screening (including body weight and temperature measurements, grip strength test, and righting, whisker touch, and ear twitch reflexes), wire hang test, Barnes maze test, eight-arm radial maze test, and fear conditioning test.

### Eight-arm radial maze test

The protocol was as previously described [42]. We used an apparatus with a central platform connected to eight arms (40-cm long with 25-cm high transparent walls, food pellet wells and sensors at distal ends) with automated shutters, which was placed 75 cm above the floor in a dim room with several spatial cues. The animals were starved for over a week to induce 15–20% weight loss and started on pre-training on the eighth day. We allowed a mouse to explore and eat food pellets for 30 min. Then, we set a pellet in a well and let a mouse explore and eat it, which was repeated eight times, once for each arm. In the spatial working memory task, we set a pellet in each well, and observed until a mouse ate the eight pellets. After each visit to an arm, the shutters were closed for 5 s with mice at the center. We video-monitored and analyzed arm choices, latency to acquire eight pellets, distance traveled, the number of times empty arms were chosen in the first eight choices, and the number of revisiting errors. Image RM software was used for the control of shutters, data acquisition and analysis (see below for ‘IMAGE ANALYSIS’).

### Contextual and cued fear conditioning test

To assess fear memory [43], mice were placed in a conditioning chamber (26 × 34 × 29 cm) in a sound attenuated room and allowed to explore freely for 2 min. The animals were presented with an auditory cue (55 dB white noise) which served as a conditioned stimulus (CS) for 30 s. During the last 2 s of the CS, mice were given a mild foot shock (2 s, 0.35 mA) as an unconditioned stimulus (US). Two more CS-US pairings were presented with 120 s interval. 24 h later, context test was performed. Cued test in an altered context was performed using a triangular box (35 × 35 × 40 cm) made of white opaque plexiglas, located in a different room. Following initial 3-min of pre-CS period, the CS was presented for 3 min. Data acquisition, control of stimuli (white noise and foot shock), and data analysis were performed automatically using Image FZ software.

### Image analysis

The application programs for behavioral data acquisition and analysis (Image BM, RM, FZ) were created on the platform of ImageJ (http://rsb.info.nih.gov/ij/) by TM.

### Antibodies

We used commercial antibodies for the following antigens: N-cadherin (clone 32) and syntaxin-6 (clone 30, BD); β-actin, PSD95, and HA (Sigma); synaptophysin (clone SY38, Abcam); ADAM10 (AB19026), Presenillin1 (MAB5232), GluA1 (MAB2263), and GLT1 (MAB2262, Chemicon/Millipore); GluA2 (#13607), AKT (#9272), phospho-AKT (#9271), and α-Tubulin (#2144, CST); mouse and rabbit IgG, HRP-conjugated (NA931V and NA931934, GE Healthcare).

### Plasmids

Ncad HA and NcadGD HA constructs, expressing the full-length human N-cadherin tagged with HA in C-terminus, as described elsewhere [20].

### Primary mouse cortical neuron culture

Primary neurons from GD mice brain were obtained from the cerebral cortices of fetal mice (14–16 days of gestation). Obtained cells were maintained in Neurobasal medium (Gibco) containing GlutaMAX™-I(Gibco), B-27 supplement (Gibco) and 1% penicillin/streptomycin (Nacalai tesque).

### Immunofluorescence

Chinese Hamster Ovary (CHO) cells were maintained in Dulbecco's Modified Eagle Medium/Nutrient Mixture DMEM/F-12 (Thermo scientific) containing 10% FBS (Invitrogen) and 1% penicillin/streptomycin (Nacalai tesque). For transient expression, Lepofectamin2000™ (Invitorogen), was used. After 24 h, CHO were fixed, permeabilized and incubated in Block Aid (Thermo) for 1 h. The primary antibodies against HA (1:1000) and β-catenin (1:1000) were incubated at 4 ℃ overnight. The secondary antibodies conjugated to Alexa-Fluor 488/546 were added for 1 h at room temperature. Cells were mounted onto slides by ProLong Gold antifade reagent with DAPI (Molecular Probes). Images were acquired using FLUOVIEW FV10i (OLYMPUS).

### Internalization assay

CHO cells were transfected with Ncad-HA(WT) or Ncad GD-HA (GD mutant) and incubated with 1 mg/ml Sulfo-NHS-LC-Biotin(Thermo) in KRPH Buffer (128 mM NaCl, 4.7 mM KCl, 1.25 mM CaCl_2_, 1.25 mM MgSO_4_, 5 mM NaH_2_PO_4_, 20 mM HEPES) at 4℃ for 1 hr. Surface-biotinylated cells were washed in PBS and washed twice more with Biotin Blocking Reagent (50 mM NH_4_Cl, 1 mM MgCl_2_, 0.1mMCaCl_2_). Cells were replaced in DMEM/F12 containing 10% FBS and incubated at 37℃ for 1,3,6,12 h. After incubation, cells were lysed in RIPA buffer (20 mM Tris–HCl pH7.4, 150 mM NaCl, 0.1% SDS, 1% TritonX-100, 1% Sodium Deoxycholate, 5 mM EDTA containing a protease inhibitor mix). Surface-biotinylated proteins were pulled down with streptavidin beads (Invitrogen), subjected to SDS PAGE and blotted with anti-HA-tag antibody (Sigma).

### Preparation of synaptosome

Synaptosome was prepared according to the previous report of P.R. DODD et al. [44]. In brief, mouse brain tissue was homogenized in 10 volumes of homogenization buffer (5 mM HEPES buffer, pH 7.4, containing 0.32 M sucrose, 50 mM sodium fluoride, with phosphatase and protease inhibitor cocktail) in a Potter–Elvehjem homogenizer. The homogenate was centrifuged at 1000 g at 4 °C for 5 min, twice. Supernatant was layered directly onto 1 ml 1.2 M sucrose and centrifuged at 50,000 rpm at 4℃ for 10 min. The Pellet of intermediate layer was collected and diluted with ice-cold 0.32 M sucrose to a final volume of 1 ml. The diluted suspension was then layered onto 0.8 ml of 0.8 M sucrose and centrifuged once again at 50,000 rpm at 4 °C for 15 min. The pellet was suspended in RIPA buffer (with protease and phosphatase inhibitors. The pellet was then layered over discontinuous sucrose gradient (0.85–1.0–1.2 M) and centrifuged at 85,000* g* for 2 h at 4 °C. Synaptosomal fraction was obtained at the interface of 1 and 1.2 M sucrose.

### Immunoblotting

Protein samples were prepared in 2 × loading buffer (0.5 M Tris (pH 6.8), 10% SDS, 12% 2-mercaptoethanol and 0.02% bromophenol blue), was separated to SDS– PAGE electrophoresis on a 5–20% precast-gels (ATTO Inc., Japan) and then transferred onto PVDF membranes. The membranes were blocked in 5% milk in TBS-T buffer (0.2 M Tris (pH7.5), 1.37 M NaCl, 1% Tween) for 1 h and then incubated with primary antibodies (1/1000) overnight. The secondary antibodies, anti-rabbit or anti-mouse IgG HRP conjugated (Cytiva) were then added for 1 h at room temperature. The membranes were washed with TBS-T, and immunoreactive bands were visualized by Chemi-Lumi One L or supper (nacalai tesque, Japan).

### Golgi staining

Golgi staining was performed using a Rapid GolgiStain kit according to the manufacturer’s protocol (FD NeuroTechnologies, Columbia,MD). Mice were sacrificed at the age of 4 months. The brains were immersed in impregnation solution for 2 weeks in the dark, and then stored at 4℃ for a week. The brain samples were dipped into isopentane pre-cooled with dry ice, mounted with TFM (TBS, Durham, Nc, USA), sliced (80 μm) with a cryostat,and then mounted on gelatin-coated glass slides. Subsequently, the sections were stained with staining solution. Measurements of the spine area were made by Image J and Metamorph (Molecular Devices).

### Serial section transmission electron microscopy (ssTEM)

The Electron microscopy procedure has been previously described [45]. Briefly, mice were fixed transcardially with 0.8% paraformaldehyde and 1.5% glutaraldehyde/0.1 M PB (pH 7.35). 70-µm sections were made with a Vibratome (VIB1500X, Leica). 50-nm ultrathin sections were cut with an ultramicrotome (Ultracut, Leica), mounted on grids and then observed with a TEM (JEM1010, JEOL).

### Electrophysiology

Mice were decapitated under deep halothane anesthesia at the age of 14 to 15 weeks, and both hippocampi were isolated. Transverse hippocampal slices (380 μm) were cut using a tissue slicer and maintained in a humidified interface holding chamber. Electro-physiological recordings were performed as described [46]. Recordings were made in a submersion-type chamber maintained at 27.0–27.5 °C and superfused at 2 ml/min with saline composed of (in mM): NaCl, 125; KCl, 2.5; NaH_2_PO_4_, 1.0; NaHCO_3_, 26.2; glucose, 11; CaCl_2_, 2.5; MgCl_2_, 1.3. For recording EPSPs arising from the A/C fiber-pyramidal cell synapses in the CA3 region, a glass recording pipette filled with 2 M NaCl and bipolar stimulating electrodes were placed in the stratum radiatum of the CA3 region. The initial slope of EPSPs was measured on analysis. Single electrical stimulation was delivered at a frequency of 0.05 Hz, unless otherwise specified. To induce LTP, high-frequency burst stimulation (5 pulse at 100 Hz, repeated 10 times at 5 Hz) was delivered 3 times at an interval of 20 s. For recording EPSPs at the Schaffer collateral/commissural synapses in the CA1 region, both recording and stimulating electrodes were placed in the stratum radiatum of the CA1 region, and the recording saline was supplemented with the GABA_A_ receptor blocker picrotoxin (100 μM) and the NMDAR antagonist D-2-Amino-5-phosphonovaleric acid (D-APV, 100 μM). Picrotoxin was purchased from FUJIFILM Wako Pure Chemical Corporation (Osaka, Japan). D-APV was from Tocris Bioscience (Bristol, UK). All recordings were made using a Multiclamp 700B amplifier (Molecular Devices, Sunnyvale, CA, USA).

### Statistical analysis

Quantitative data were expressed as mean ± SEM. For statistical analyses, either two tailed t-test or ANOVA (one-way, two-way repeated measures, and repeated measures with two factors) was applied using StatView (SAS institute). F and p values represent the effects of genotype, unless otherwise noted.

Results of behavioral analysis was summarized in Additional file 1: Table S1. When we found significant difference in genotype and genotype × Trial/Delay/Time interaction (p < 0.05), the data were processed with Dunn-Bonferroni post hoc test.

## Supplementary Information

**Additional file 1: Fig. S1** General behavioral characteristics of GD mice. (A) Body weight (g), (B) body temperature (°C), (C) grip strength (Newton, N) (D) and wire hang latency (s) are represented. The p-values indicate genotype effect in one-way ANOVA. Values are means ± SEM. Suppl. **Fig. S2.** Barnes maze in GD mice. (A) Latency, (B) Number of errors, and (C) Distance traveled to first reach the target hole during the training session. The p-values indicate genotype effect in two-way repeated measures ANOVA. Values are means ± SEM. The test was conducted on a white circular surface, 1.0 m in diameter, with 12 holes equally spaced around the perimeter (O’Hara & Co., Tokyo, Japan). The circular open field was elevated 75 cm from the floor. A black Plexiglas escape box (17 × 13 × 7 cm), which had paper cage bedding at the bottom, was located under one of the holes. The hole above the escape box represented the target. The maze was rotated daily, with the spatial location of the target unchanged with respect to the distal visual room cues. Three trials per day were conducted for 5 days. One day and 8 days after the acquisition test, probe trials were conducted without the escape box. The number of errors, latency and distance traveled to reach the target hole and the time spent around each hole were recorded by Image BM software. **Fig. S3.** Probe test of Barnes maze in GD mice. (A) Time spent around the target hole in the probe test 1 day after the last training session (right panel). Latency, number of errors, and distance traveled to first reach the target hole are represented in left panel. (B) Time spent around the target hole in the probe test 8 days after the last training session (right panel). Latency, number of errors, and distance traveled to first reach the target hole are represented in left panel. The p-values indicate genotype effect in two-way repeated measures ANOVA (A; left panel, B; left panel) or one-way ANOVA (A; three panels on the right, B; three panels on the right). Values are means ± SEM. **Fig. S4.** Brain weight and macroscopic architecture in GD mice. There is no obvious difference in the total weight (A) and macroscopic architecture (B) of the brain between WT and GD mouse (n = 3, p = 0.4871, t-test). (C) Golgi staining of spines in CA1 region. There is no obvious difference in CA1 region between WT and GD mouse brains. **Fig. S5**. Detection of trans-dimer in WT or GD mutant N-cadherin. (A) Scheme of the experiment methods to detect N-cadherin trans-dimer. Either NcadWT(GD)-HA or NcadWT(GD)-Flag were separately transfected into HEK293 cells. 24 after transfection, cells were scraped off. Then HEK293 cells transfected with NcadWT(GD)-HA and with NcadWT(GD)-Flag were co-cultured for another 48hrs to allow formation of trans-dimer (dimer formed by cell–cell adhesion). Cells were harvested and N-cadherin trans-dimer were detected by immunoprecipitation with anti-HA antibody followed by western blot. (B) Cell lysates were pulled down with anti-HA anti-body, followed by the blotting with anti-Flag antibody. (C) N-cadherin trans-dimer quantified by normalization with total N-cadherin and β-actin, mean + SD (p = 0.8274 n = 4, t-test). **Fig. S6**. Intact basal synaptic transmission in hippocampal CA1 region of GD mice. Schaffer collateral/commissural fiber-CA1 pyramidal cell synaptic transmission was examined in the presence of the NMDAR antagonist D-APV. GD mice showed no changes in the dependence of fiber volley amplitude (left) and EPSP slope (right) on stimulus intensity. **Table S1**.

## Data Availability

Data sharing not applicable to this article as no datasets were generated or analyzed during the current study. For the request of genetically modified mouse strains, please contact the author.

## Abbreviations

NMDAR: N-Methyl-d-Aspartate receptor
ADAM10: A-disintegrin and metalloprotease 10
PSD: Postsynaptic density
AMPA: α-3-Hydroxy-5-methyl-4-isoxazole propionic acid
HA: Human influenza hemagglutinin
CHO: Chinese hamster ovary
ANOVA: Analysis of variance
cx: Cerebral cortex
KI: Knock-in
wt: Wild-type
