# Supplementary material for: Mice with cleavage-resistant N-cadherin exhibit synapse anomaly in the hippocampus and outperformance in spatial learning tasks
Source: Mol Brain. 2021 Jan 25;14:23. doi: 10.1186/s13041-021-00738-1 (PMC7831172; doi:10.1186/s13041-021-00738-1)
Supplement: Supplementary file 1 — Additional file 1: Fig. S1 General behavioral characteristics of GD mice. (A) Body weight (g), (B) body temperature (°C), (C) grip strength (Newton, N) (D) and wire hang latency (s) are represented. The p-values indicate genotype effect in one-way ANOVA. Values are means ± SEM. Suppl. Fig. S2. Barnes maze in GD mice. (A) Latency, (B) Number of errors, and (C) Distance traveled to first reach the target hole during the training session. The p-values indicate genotype effect in two-way repeated measures ANOVA. Values are means ± SEM. The test was conducted on a white circular surface, 1.0 m in diameter, with 12 holes equally spaced around the perimeter (O’Hara & Co., Tokyo, Japan). The circular open field was elevated 75 cm from the floor. A black Plexiglas escape box (17 × 13 × 7 cm), which had paper cage bedding at the bottom, was located under one of the holes. The hole above the escape box represented the target. The maze was rotated daily, with the spatial location of the target unchanged with respect to the distal visual room cues. Three trials per day were conducted for 5 days. One day and 8 days after the acquisition test, probe trials were conducted without the escape box. The number of errors, latency and distance traveled to reach the target hole and the time spent around each hole were recorded by Image BM software. Fig. S3. Probe test of Barnes maze in GD mice. (A) Time spent around the target hole in the probe test 1 day after the last training session (right panel). Latency, number of errors, and distance traveled to first reach the target hole are represented in left panel. (B) Time spent around the target hole in the probe test 8 days after the last training session (right panel). Latency, number of errors, and distance traveled to first reach the target hole are represented in left panel. The p-values indicate genotype effect in two-way repeated measures ANOVA (A; left panel, B; left panel) or one-way ANOVA (A; three panels on the right, B; three pan [file 13041_2021_738_MOESM1_ESM.pptx]

## Slide 1
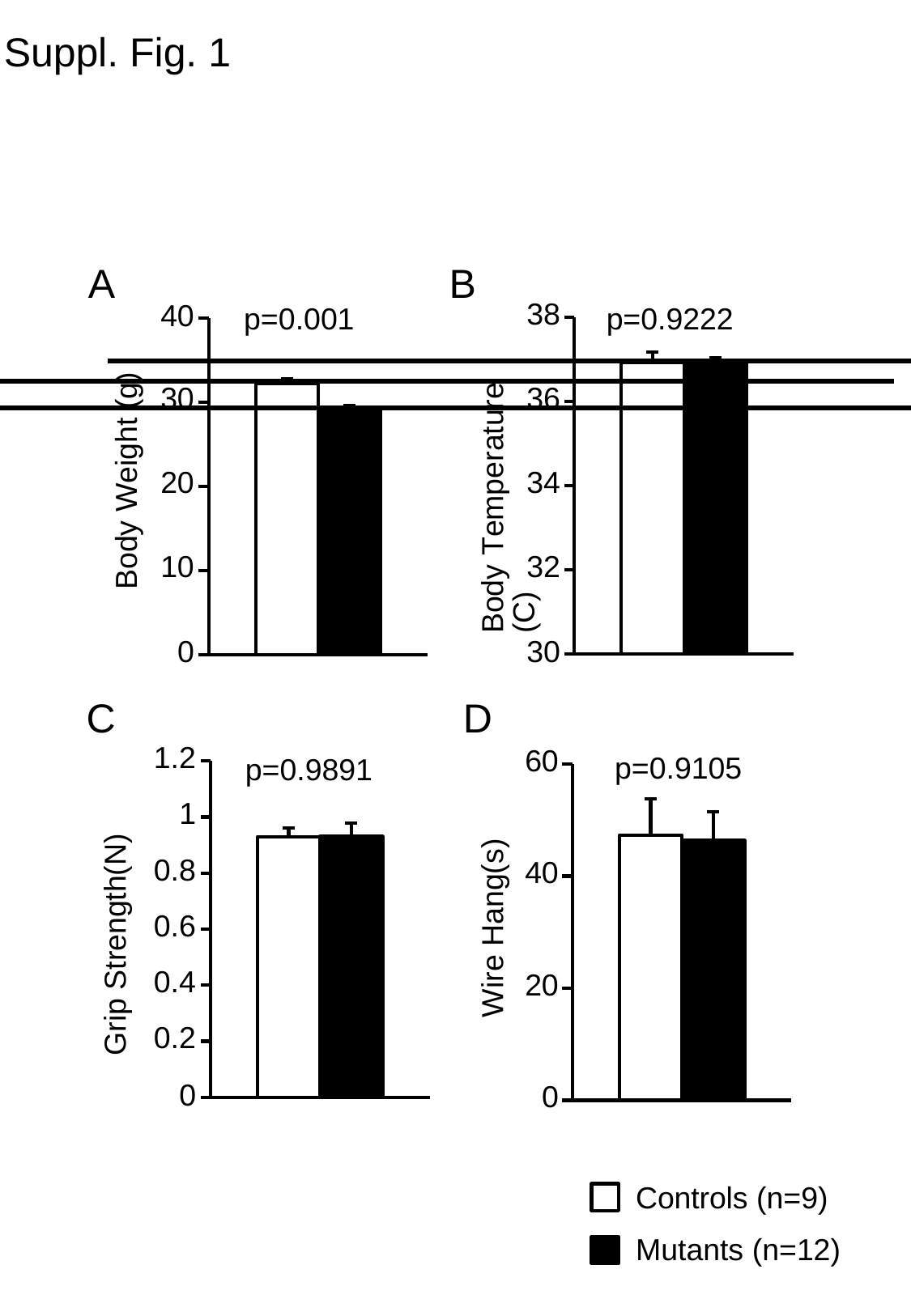

Suppl. Fig. 1
A
B
38
40
p=0.001
p=0.9222
Body Temperature (C)
Body Weight (g)
30
36
20
34
10
32
0
30
D
C
1.2
1
60
p=0.9105
p=0.9891
Grip Strength(N)
Wire Hang(s)
0.8
0.6
0.4
0.2
40
20
0
0
Controls (n=9)
Mutants (n=12)

## Slide 2
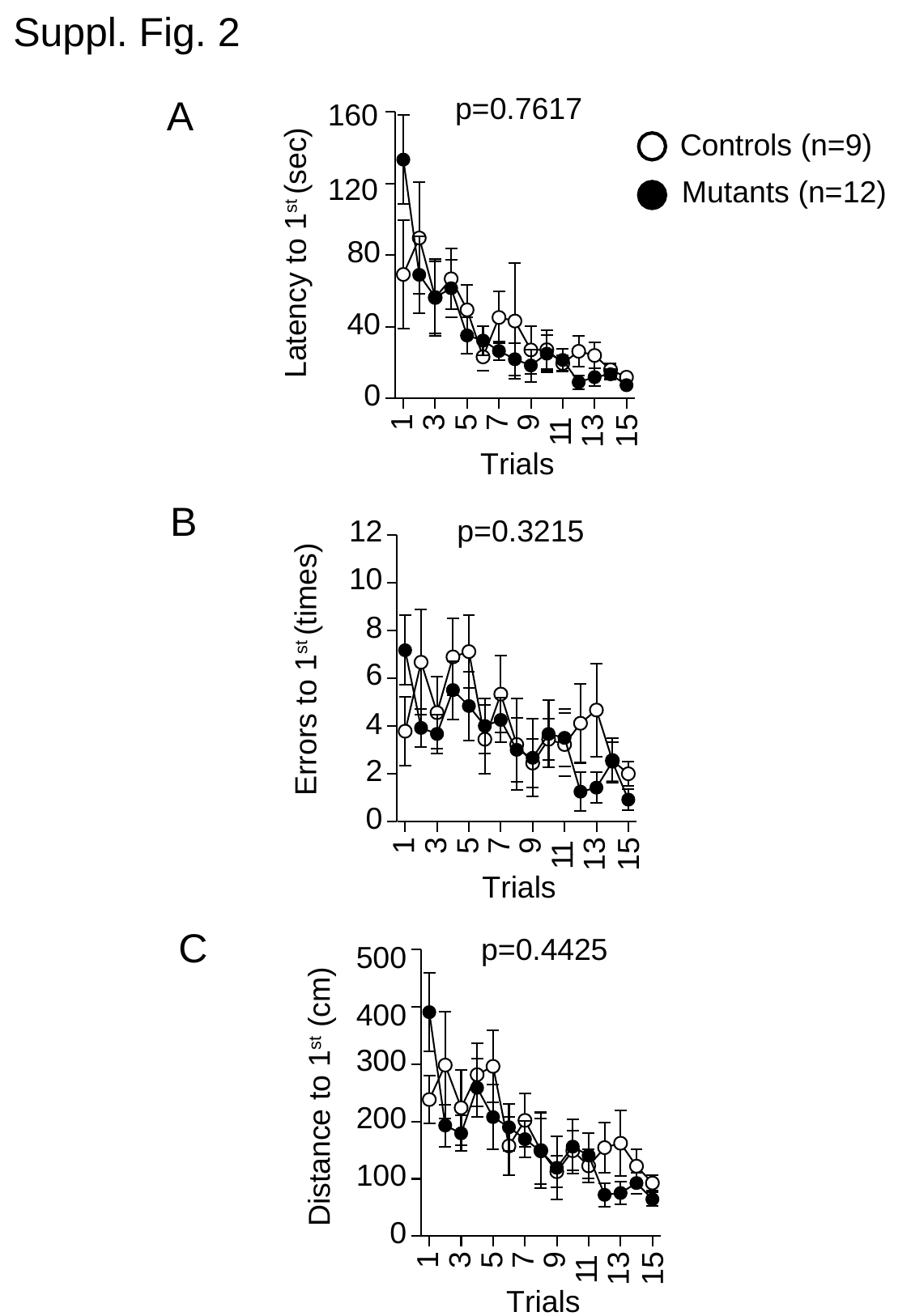

Suppl. Fig. 2
160
120
A
p=0.7617
Latency to 1st (sec)
Controls (n=9)
Mutants (n=12)
80
40
0
1
3
5
7
9
11
13
15
Trials
Errors to 1st (times)
B
12
10
8
6
4
2
0
p=0.3215
1
3
5
7
9
11
13
15
Trials
500
400
C
p=0.4425
Distance to 1st (cm)
300
200
100
0
1
3
5
7
9
11
13
15
Trials

## Slide 3
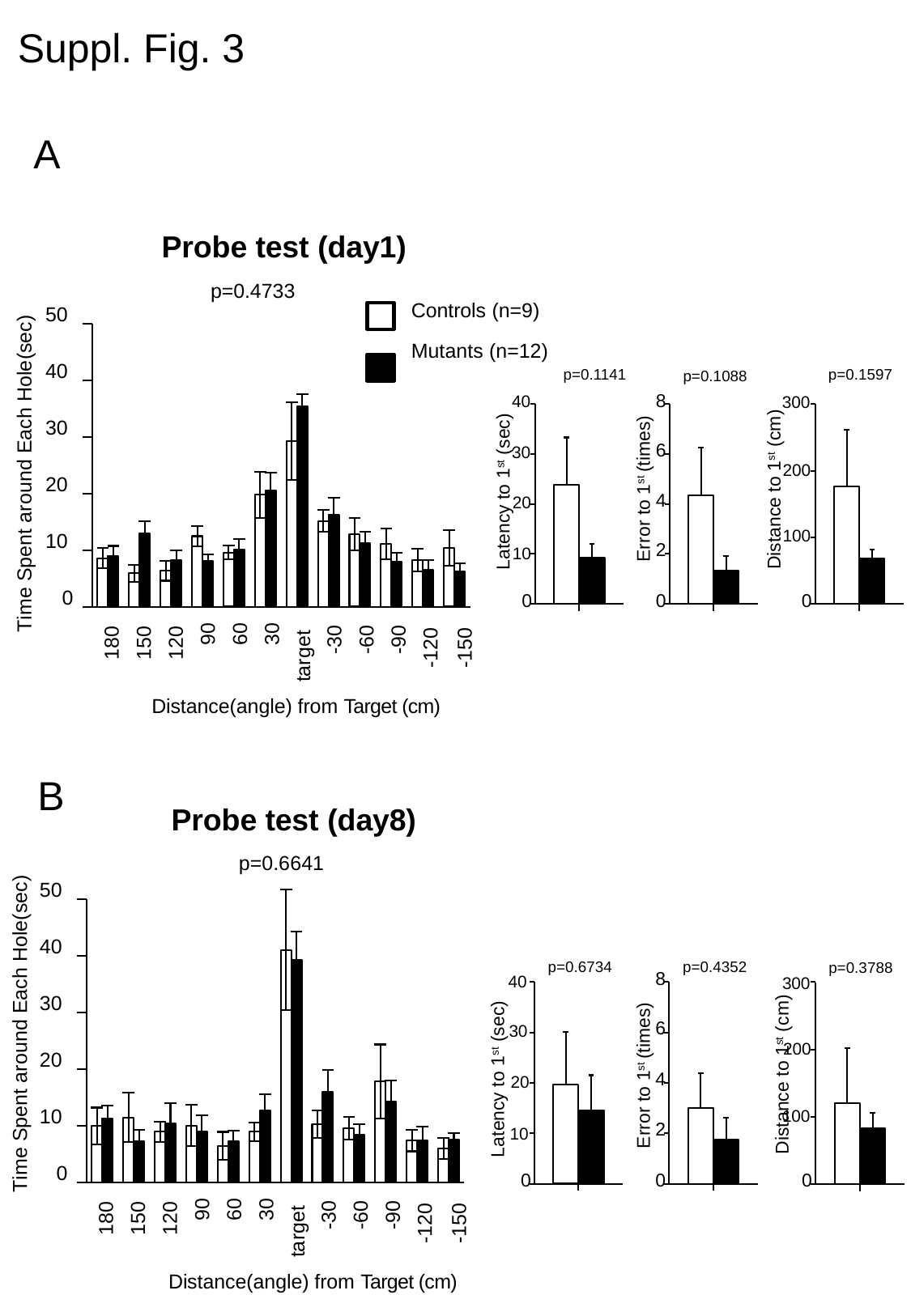

Suppl. Fig. 3
A
Time Spent around Each Hole(sec)
Probe test (day1)
p=0.4733
Controls (n=9)
Mutants (n=12)
50
p=0.1141
p=0.1597
p=0.1088
8
Distance to 1st (cm)
40
300
Error to 1st (times)
Latency to 1st (sec)
6
30
200
4
20
100
2
10
0
0
0
40
30
20
10
0
180
150
120
90
60
30
target
-30
-60
-90
-120
-150
Distance(angle) from Target (cm)
Time Spent around Each Hole(sec)
B
Probe test (day8)
p=0.6641
50
40
p=0.4352
p=0.6734
p=0.3788
8
40
300
Distance to 1st (cm)
Error to 1st (times)
Latency to 1st (sec)
6
30
200
4
20
100
2
10
0
0
0
30
20
10
0
180
150
120
90
60
30
target
-30
-60
-90
-120
-150
Distance(angle) from Target (cm)

## Slide 4
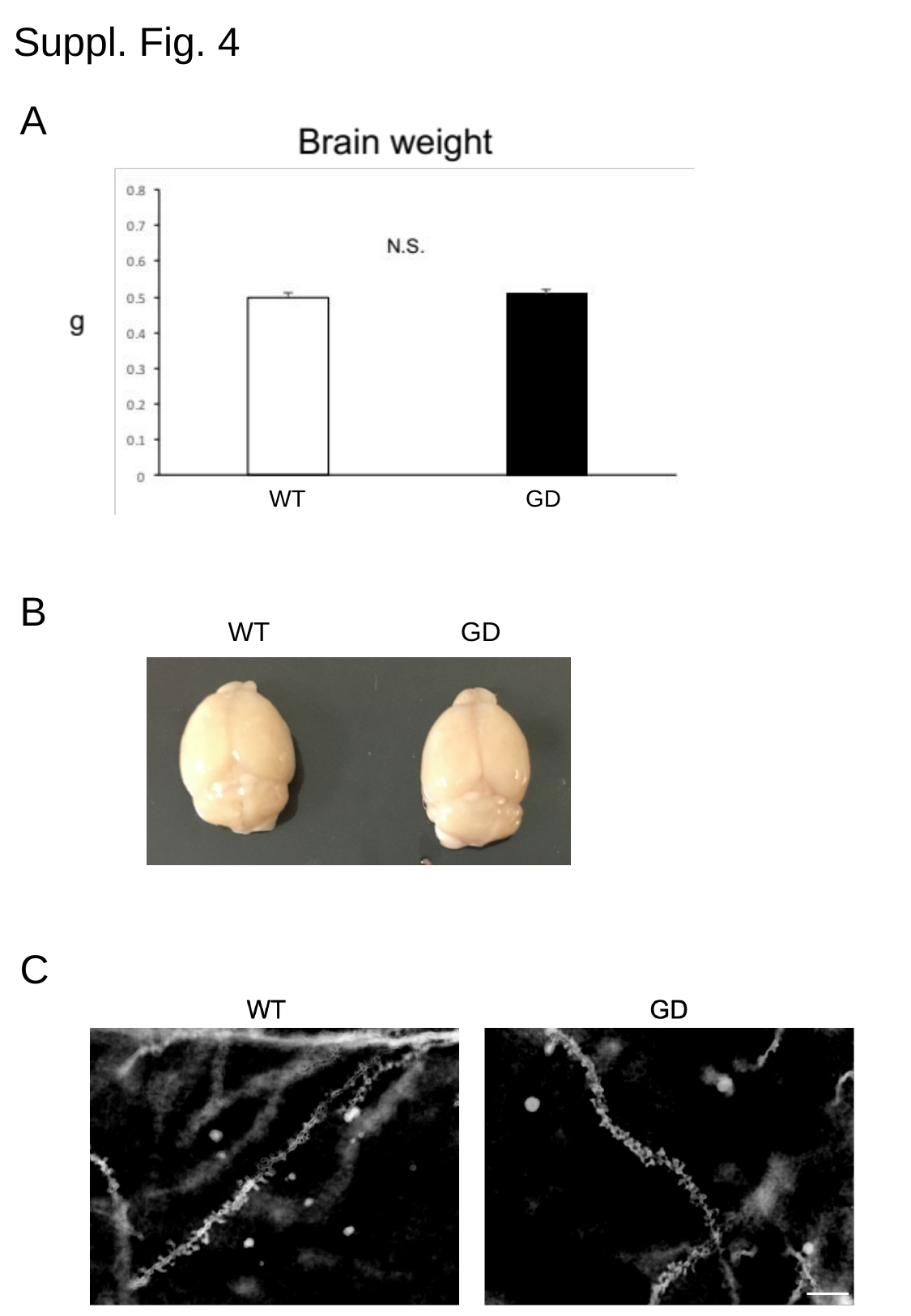

Suppl. Fig. 4
A
WT
GD
B
WT
GD
C

## Slide 5
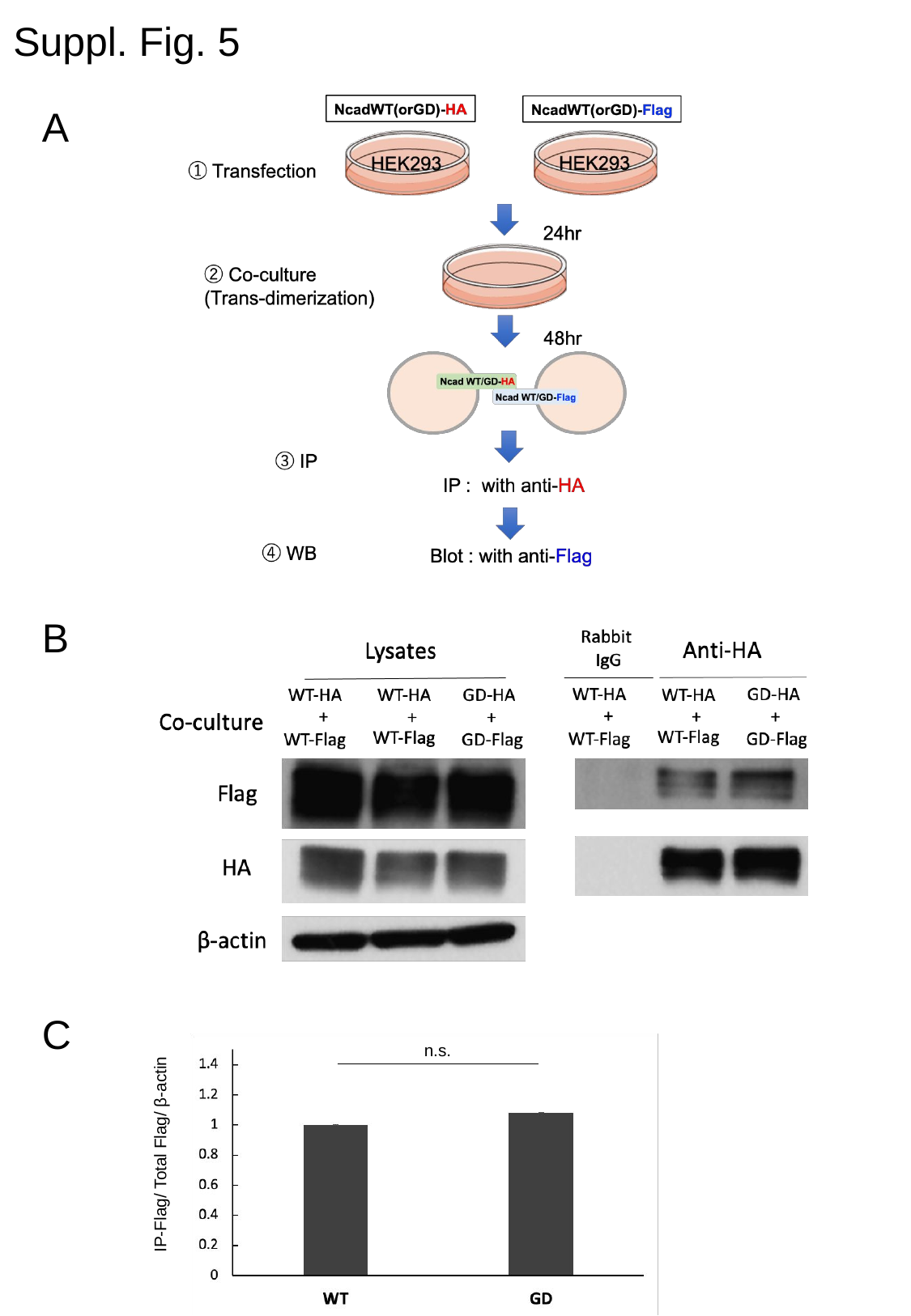

Suppl. Fig. 5
A
B
C
n.s.
IP-Flag/ Total Flag/ β-actin

## Slide 6
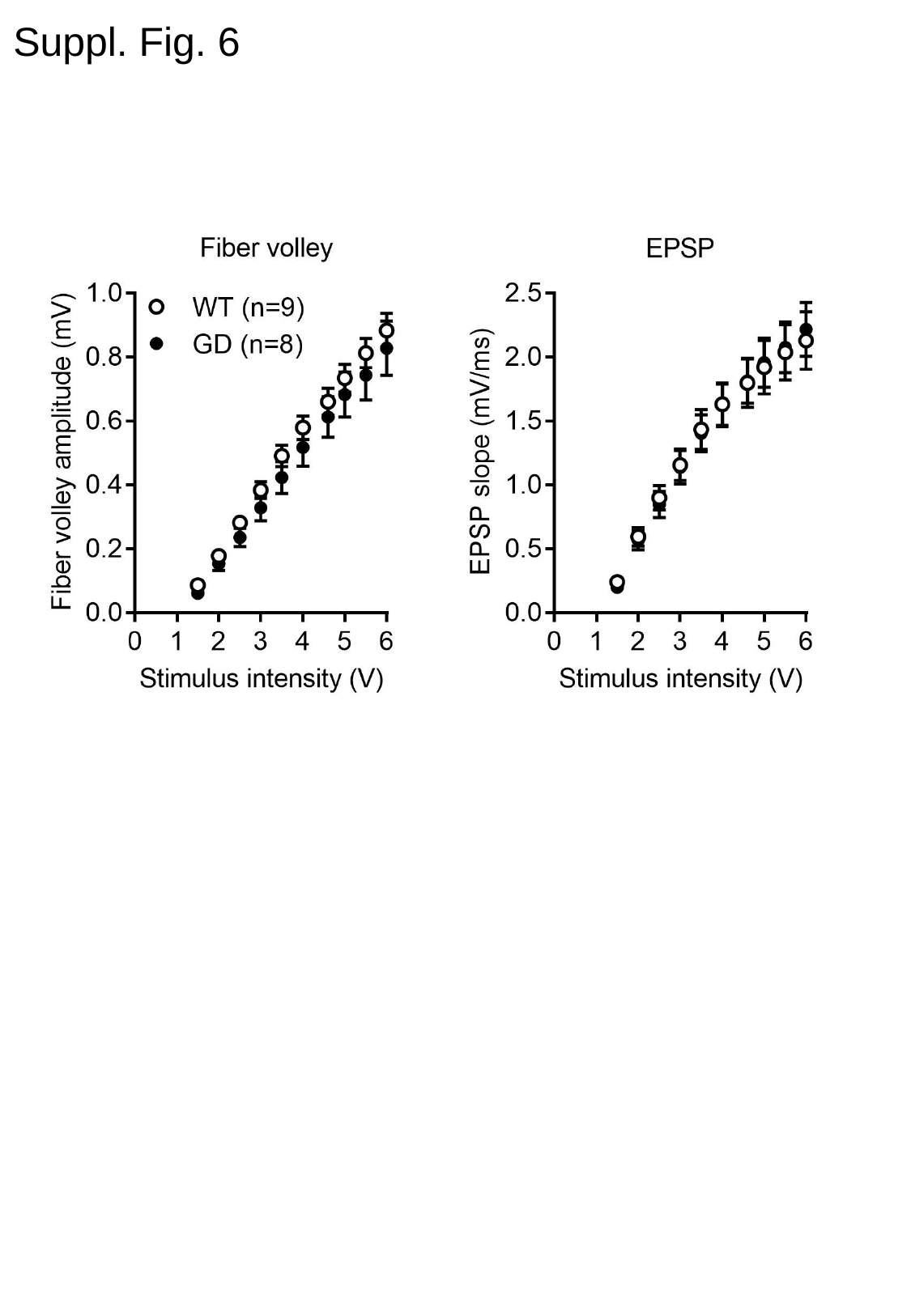

Suppl. Fig. 6

## Slide 7
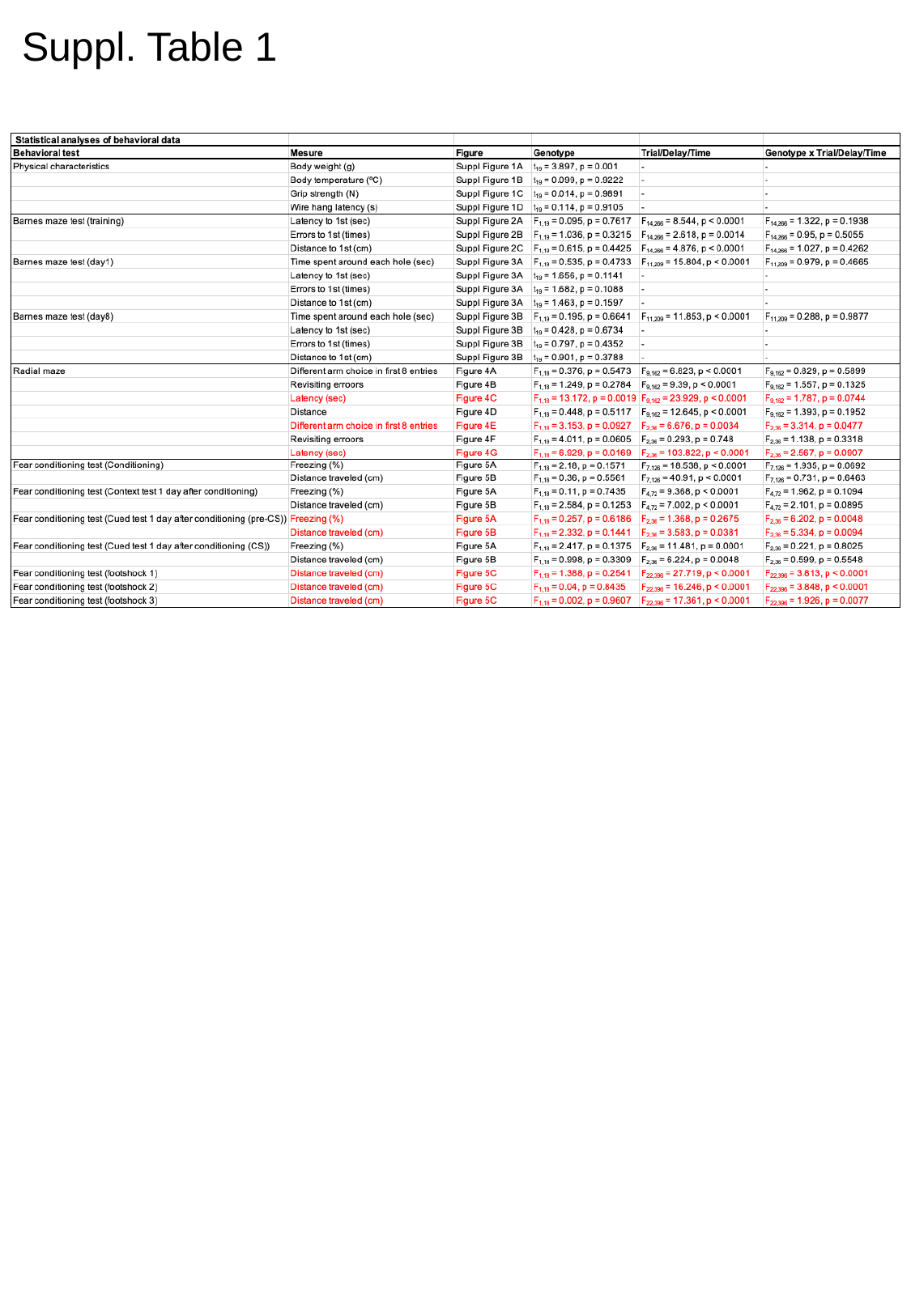

Suppl. Table 1
